# Impaired Expression of Cytoplasmic Actins Leads to Chromosomal Instability of MDA-MB-231 Basal-Like Mammary Gland Cancer Cell Line

**DOI:** 10.3390/molecules26082151

**Published:** 2021-04-08

**Authors:** Vera Dugina, Galina Shagieva, Mariya Novikova, Svetlana Lavrushkina, Olga Sokova, Igor Kireev, Pavel Kopnin

**Affiliations:** 1 Belozersky Institute of Physico-Chemical Biology, Lomonosov Moscow State University, 119992 Moscow, Russia; vdugina@iname.com (V.D.); galya_shagieva@yahoo.com (G.S.); blodemwold@gmail.com (S.L.); kireev@genebee.msu.ru (I.K.); 2 Biological Faculty, Lomonosov Moscow State University, 119234 Moscow, Russia; 3 N.N. Blokhin National Medical Research Center of Oncology, 115478 Moscow, Russia; mvnovikova94@mail.ru (M.N.); flesok@yandex.ru (O.S.)

**Keywords:** mammary gland cancer, actin isoforms, chromosomal instability (CIN)

## Abstract

We have shown previously that two cytoplasmic actin isoforms play different roles in neoplastic cell transformation. Namely, β-cytoplasmic actin acts as a tumor suppressor, whereas γ-cytoplasmic actin enhances malignant features of tumor cells. The distinct participation of each cytoplasmic actin in the cell cycle driving was also observed. The goal of this study was to describe the diverse roles of cytoplasmic actins in the progression of chromosomal instability of MDA-MB-231 basal-like human carcinoma cell line. We performed traditional methods of chromosome visualization, as well as 3D-IF microscopy and western blotting for CENP-A detection/quantification, to investigate chromosome morphology. Downregulation of cytoplasmic actin isoforms alters the phenotype and karyotype of MDA-MB-231 breast cancer cells. Moreover, β-actin depletion leads to the progression of chromosomal instability with endoreduplication and aneuploidy increase. On the contrary, γ-actin downregulation results not only in reduced percentage of mitotic carcinoma cells, but leads to chromosome stability, reduced polyploidy, and aneuploidy.

## 1. Introduction

Actin and actin-binding proteins regulate the functional state of chromatin and gene expression, and, apparently, they play an important role in the organization of the genome [1]. Previous studies have demonstrated important participation of actin and its regulatory proteins in cell division [2]. We have shown clear segregation of cytoplasmic actin isoforms in anaphase-telophase of normal mitotic epithelial cells [3,4], and that depletion of each cytoplasmic actin led to impaired proliferation/cell cycle of carcinoma cells [5,6]. The distinct roles of β- and γ-actins in the cell cycle were observed: only downregulation of β-actin induced a significant decrease in diploid cell population and accumulation of near-tetraploid cells, which was detected by flow cytometry. There was no effective inhibition of proliferation with a decrease in the expression of β-actin in MDA-MB-231 cells. Moreover, for this cell type β-actin depletion led to an obvious increase in the number of cells in G2/M phase. An enrichment of aneuploid cell population was detected in β-actin depleted MDA-MB-231 cells by flow cytometry. It should be noted that this method did not permit to characterize exact DNA content and chromosome numbers. Here we specified the karyotype of MDA-MB-231 cell line, to investigate the influence of actin’s expression on chromosome organization. Many data suggest that aneuploidy often occurs due to a certain type of genetic instability, chromosomal instability (CIN), which may indicate defects in mitotic segregation in cancer cells [7].

To investigate chromosome heterogeneity and instability, traditional methods of chromosome visualization, as well as 3D-IF microscopy for CENP-A detection and quantification, were performed. The majority of human CENP-A is located at centromeric sites in the homotypic chromatin at all the cell cycle points [8]. Dysfunctions in centromere chromatin lead to chromosomal instability. Chromosomal instability is considered to be one of the major causes of genomic instability and cancer progression. Therefore, mechanisms for assembling, maintaining, and regulating centromere chromatin are crucially important in assuring proper chromosome segregation and avoiding chromosomal/genomic instability [9].

Downregulation of cytoplasmic actin isoforms alters the phenotype and karyotype of MDA-MB-231 cells. Moreover, β-actin depletion leads to the progression of chromosomal instability.

## 2. Results

### 2.1. Downregulation of Cytoplasmic Actin Isoforms Alters the Phenotype of MDA-MB-231 Cell Line

**Figure 1:**
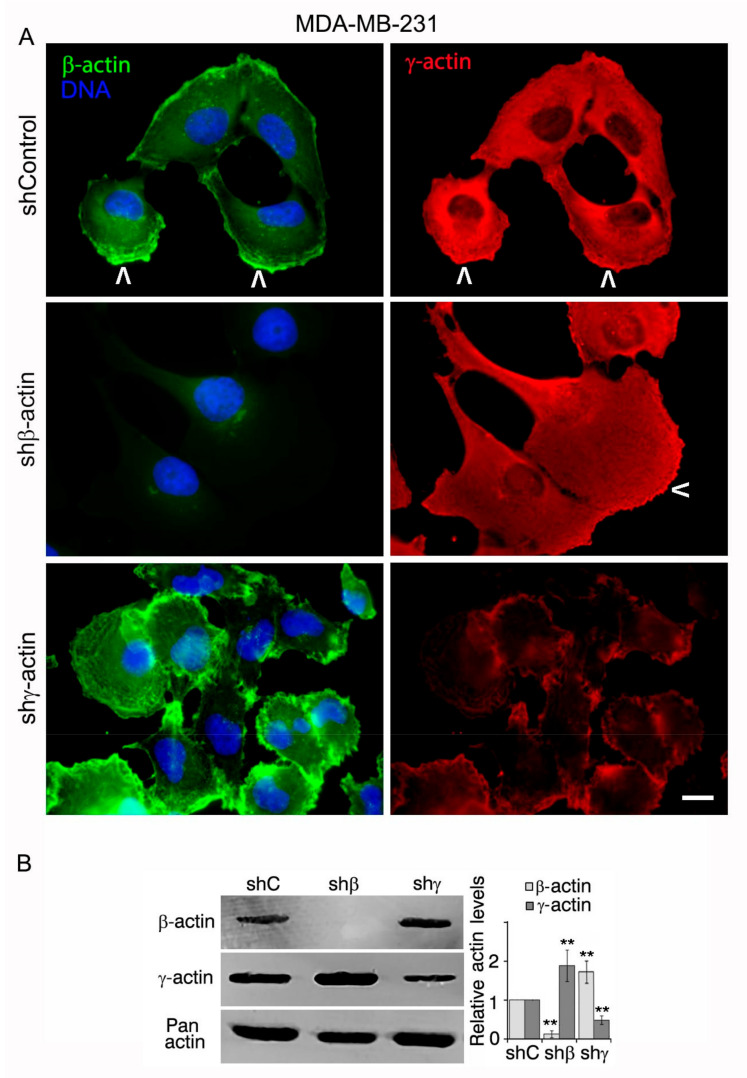
Downregulation of β- or γ-actin expression by corresponding shRNAs in MDA-MB-231 breast cancer cells leads to phenotype changes. (**A**). Immunofluorescent staining of MDA-MB-231 cells with β- or γ-actin downregulation by corresponding shRNAs. Arrowheads mark leading edge with actin enrichment. Bar, 10 µm. (**B**). Downregulation of cytoplasmic β- or γ-actin in MDA-MB-231 cells. WB analysis. Graphs represent relative actins expression (Mean ± SEM). For cells with shRNA compared with control (β-actin): *p* = 0.0015 (shRNA to β-actin); *p* = 0.007 (shRNA to γ-actin); (γ-actin): *p* = 0.0037 (shRNA to β-actin); *p* = 0.0048 (shRNA to γ-actin); Mann–Whitney U test, n = 3. Values of *p* < 0.01 (**) were considered as statistically significant.

To study the individual impact of actin isoforms on chromosome heterogeneity and instability, we obtained MDA-MB-231 (Figure 1A) derivatives with silenced β- and γ-actins. shRNAs depletions were effective and specific for each cytoplasmic actin isoform at 6d of infection (Figure 1A,B). It is important to mention that a shift of the amount of one isoform is compensated by reciprocal change of another isoform: β-actin strong depletion was accompanied by γ-actin upregulation; γ-actin downregulation resulted in an increase of β-actin expression in MDA-MB-231 and other cells [5]. The total actin amount remains unchanged despite β- or γ-actin overexpression or silencing. The compensatory mechanism for cytoplasmic actin isoforms expression acts both on mRNA and protein levels and is supposed to be universal for different cell types [5,10,11,12,13].

IF staining of parental MDA-MB-231 culture was mainly presented by disorganized or nearly diffuse β-actin with concentration at the leading edge and evenly distributed cortical γ-actin (Figure 1A). Almost complete β-actin downregulation induced more fibroblast-like cell phenotype with an enrichment of γ-actin staining at the leading edge of MDA-MB-231 cells. γ-Actin reduction induced more epithelial morphology (Figure 1A). 

### 2.2. Downregulation of Cytoplasmic Actin Isoforms Induces Cell Cycle Alterations in MDA-MB-231 Cells 

**Figure 2:**
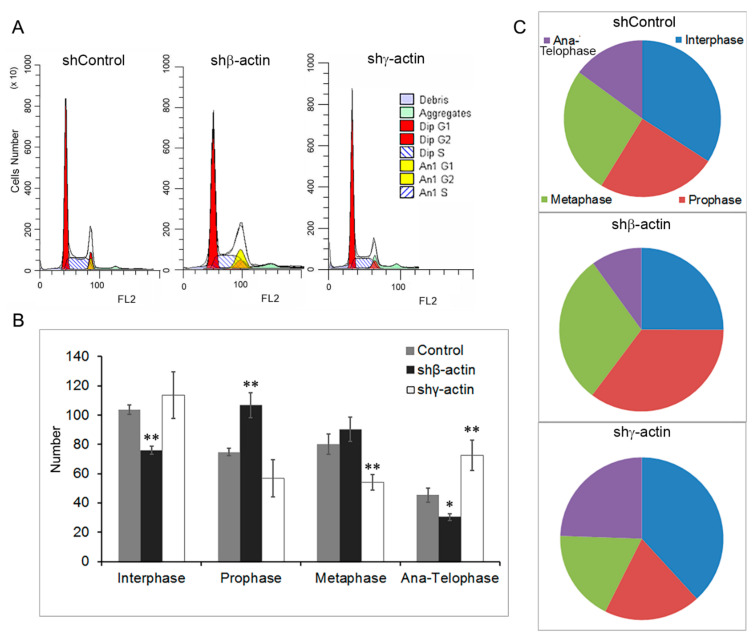
Downregulation of β- or γ-actin expression by corresponding shRNAs in MDA-MB-231 breast cancer cells leads to cell cycle changes. (**A**). After 6d of β- or γ-actin depletion by corresponding shRNAs MDA-MB-231 cells were stained with propidium iodide. High content analysis of cell cycle distributions of flow cytometric data was performed after 6d of β- or γ-actin depletion by corresponding shRNAs in MDA-MB-231 cells. (**B**). The effects of β- or γ-actin depletion on quantitative ratio of cells in interphase and different mitotic phases were analyzed by IF cytometry. The *Y*-axis indicates the number of cells in specific phases. Student’s *t*-test, n = 3. Values of *p* < 0.01 (**), and *p* < 0.05 (*) were considered as statistically significant. (**C**). The effects of β- or γ-actin depletion on mitotic phases were analyzed by IF cytometry. The pie chart shows the proportion of cells in different phases of mitosis.

We have previously shown that silencing of γ-actin or β-actin in different carcinoma cell lines led to significant alterations in proliferation in vitro [5,6]. Nonetheless, there was no effective inhibition of proliferation with a decrease in the expression of β-actin in MDA-MB-231 mammary gland carcinoma cells. To determine the effect of actin’s downregulation on the cell cycle of MDA-MB-231 cells, we used flow cytometry after 6d of actin’s downregulation, where an influence of the treatment was evident on the level of protein (Figure 2B). Cell cycle analysis using a high-content system showed that in control MDA-MB-231 cells β-actin depletion led to an obvious increase in the number of cells in G2/M phase from 37.5 ± 8,5% (sh-control) to 56.6 ± 10.5% (Figure 2A). In addition to diploid cells, aneuploid cells were detected in sh-control (6.8 ± 4.6%) and in β-actin depleted MDA-MB-231 cells (15.1 ± 7.3%). For MDA-MB-231 cells γ-actin depletion led to an obvious decrease in the number of cells in G2/M phase (Figure 2A) and an increase in G1 phase.

We have also analyzed the mitotic phases in MDA-MB-231 cultures at the same treatment conditions (6d of actin’s downregulation) by IF cytometry (Figure 2B,C). β-Actin depleted MDA-MB-231 cells with reciprocally elevated γ-actin (see above) had an enrichment of prophase/metaphase population compared with control, whereas γ-actin downregulation induced an enrichment of cell proportion at telophase and interphase stages and reduced prophase/metaphase stage compared with control (Figure 2B,C). We show here that in MDA-MB-231 basal-like breast cancer cell culture β-actin depletion not only targets cell cycle regulation but increases aneuploidy. Taken together, the above results indicated that β-actin depletion with γ-actin upregulation correlated with G2/M phase accumulation, whereas γ-actin downregulation caused G0/G1 arrest of cell cycle. 

### 2.3. Karyotype Analysis of MDA-MB-231 Cell Line

**Figure 3:**
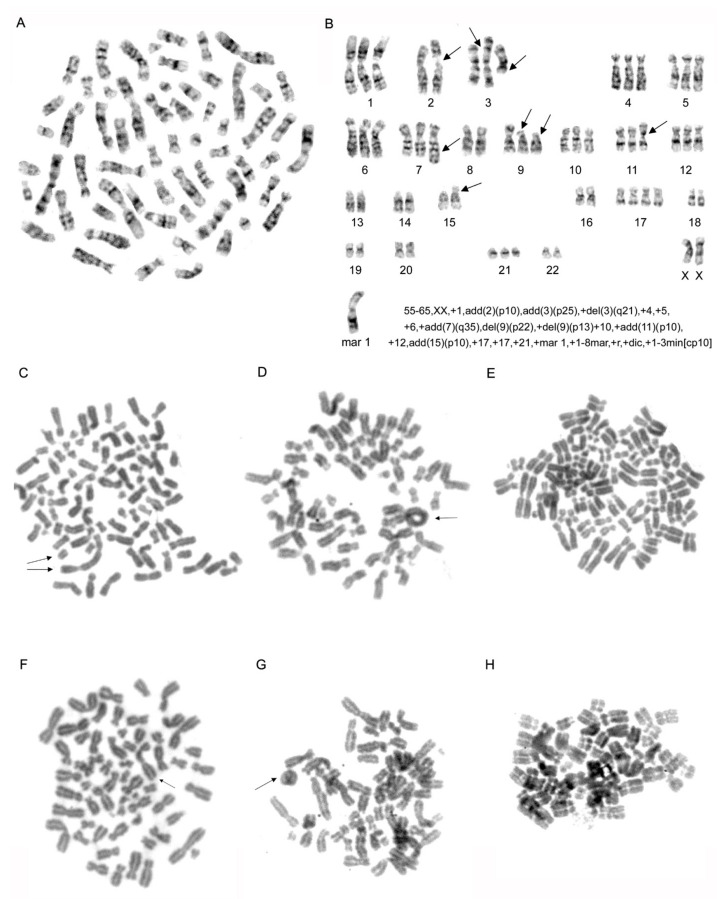
Karyotypic analysis of MDA-MB-231 breast cancer cells. (**A**). A representative G-banded karyotype of MDA-MB-231 cell line. (**B**). Composite G-banded karyotype of the near-triploid cell line MDA-MB-231, showing structural and numerical changes. Arrows point to main chromosomal alterations. Mar—Marker chromosome. (**C**,**D**,**F**,**G**). The representative chromosome aberrations in β-actin (**C**,**D**) and γ-actin (**F**,**G**) -depleted MDA-MB-231 cells: dicentric chromosomes (**C**,**F**) and acentric fragment (**C**), ring chromosomes (**D**,**G**). Arrows point to aberrations. E, H. Endoreduplication in β-actin (**E**) and γ-actin (**H**) -depleted MDA-MB-231 cell.

We specified the karyotype of MDA-MB-231 cell line using classical methods of chromosome visualization. Analysis of chromosome number and structure in 200 mitotic cells and chromosome G-banding in 20 metaphase cells revealed that cytogenetic features of our MDA-MB-231 line correspond to the data presented on ATCC (American Type Culture Collection) resource [14,15]. This is supported by hypo-triploid set of chromosomes with modal class 55–65 and several specific markers: add (2)(p10), del(3)(q21) del(9)(p22), add(15)(p10) and specific marker chromosome (Figure 3A,B; Figure S1). 

Analysis of cell populations with downregulation of actin expression revealed that in 200 metaphases were 10% of polyploids (20 per 200) in the cells with downregulation of β-actin, 4% (8 per 200) in the cells with downregulation of γ-actin, and 3.5% (7 per 200) in the control cells. We analyzed the number of chromosomes in 200 metaphase cells after shRNA treatment. We identified the differences of chromosome number and modal class in MDA-MB-231 cells after downregulation of cytoplasmic actin isoforms compared with control cells. In the cells with abnormal metaphases in both control and actin-downregulated MDA-MB-231 lines only chromosomal aberrations were detected (Figure 3C–H). There were no statistically significant differences in the ring (Figure 3C,F) and dicentric (Figure 3D,G) chromosome quantity in different experimental conditions of actin isoforms depletion. Small amount of endomitoses were found by a classical karyotype method in both β- and γ-actin suppressed cells, but β-actin downregulation led to increase of endoreduplication (Figure 3E,H). Karyotypic analysis showed different aneuploidies from 60 to 120 chromosomes and variable chromosomal rearrangements in the analyzed subline with β-actin downregulation. 

### 2.4. Analysis of CENP-A, Histone H3, and Phospho-Histone H3 in MDA-MB-231 Cells after Downregulation of Cytoplasmic Actins

**Figure 4:**
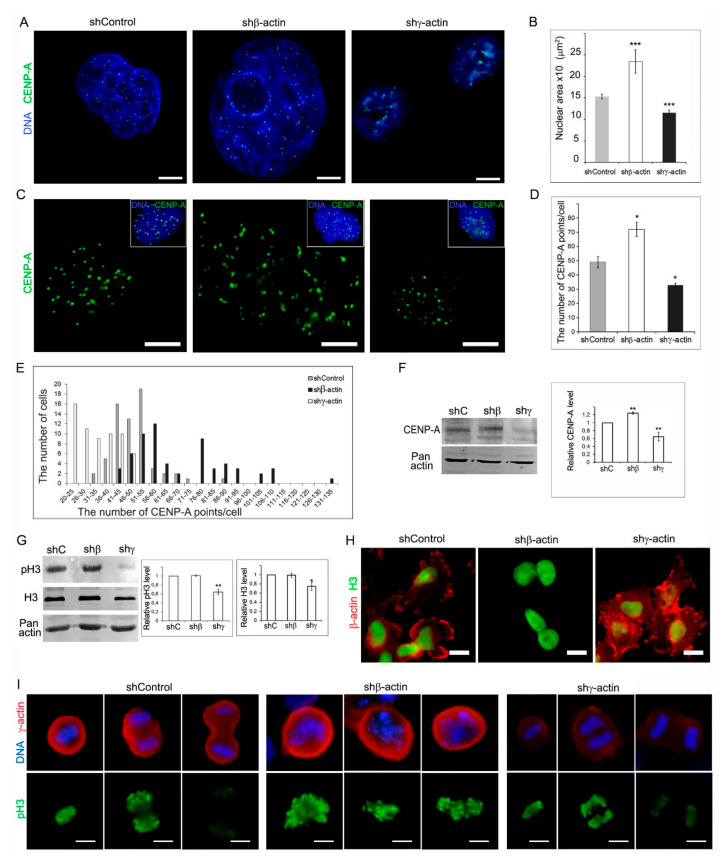
Chromosome stability is regulated by β-/γ-actins level in MDA-MB-231 breast cancer cells. (**A**). Nuclear localization of centromere-associated protein CENP-A (green) in interphase of MDA-MB-231 cells with downregulated β- or γ-actin; DNA staining by DAPI (blue). Immunofluorescence staining and high-resolution wide-field microscopy visualization, maximum intensity projection. Bar 5 μm. (**B**). Graph represents the quantification of nuclear area in MDA-MB-231 cells with downregulated β- or γ-actin (Mean ± SEM). For cells with shRNA compared with control (nuclear area): *p* = 0.00012 (shRNA to β-actin), *p* = 0.00016 (shRNA to γ-actin), Student’s t-test, n = 5. Values of *p* < 0.001 (***), *p* < 0.01 (**), and *p* < 0.05 (*) were considered as statistically significant. (**C**). Visualization of CENP-containing nucleosome particles (points) in MDA-MB-231 cell with downregulated β- or γ-actin. Immunofluorescence staining of CENP-A (green), and DNA (DAPI, blue). CENP-A particles are shown in the panels (bar 5 μm) and CENP-A with DAPI (in the frame). (**D**). Graph represents the quantification of CENP-A particles in MDA-MB-231 cells with downregulated β- or γ-actin. The Y-axis indicates the number of CENP-A points/cell in different MDA-MB-231 sub-lines with downregulated β- or γ-actin (Mean ± SEM). For cells with shRNA compared with control (CENP-A): *p* = 0.014 (shRNA to β-actin); *p* = 0.023 (shRNA to γ-actin), Student’s t-test, n = 3. (**E**). The quantitative distribution of cells with different number of CENP-A points/cell in different MDA-MB-231 sub-lines with downregulated β- or γ-actin. (**F**). WB analysis of CENP-A expression in MDA-MB-231 cells with downregulated β- or γ-actins. For cells with shRNA compared with control (CENP-A): *p* = 0.0007 (shRNA to β-actin); *p* = 0.0056 (shRNA to γ-actin), Mann–Whitney U test, n = 4. (**G**). WB analysis of H3 and p-H3 expression in MDA-MB-231 cells with downregulated β- or γ-actins. For cells with shRNA to γ-actin compared with control: p (p-H3) = 0.0038; p (H3) = 0.0028, Mann–Whitney U test, n = 3. (**H**). Visualization of H3 histone in MDA-MB-231 cells with downregulated β- or γ-actin. Immunofluorescence staining of H3 (green), β-actin (red) and DNA (DAPI staining, blue). Bar, 10 μm. (**I**). Visualization of pH3 histone in mitotic MDA-MB-231 control cells (metaphase, anaphase, telophase), with downregulated β-actin (prometa-metaphase) and with downregulated γ-actin (metaphase, anaphase, telophase). Immunofluorescence staining of H3 (green), γ-actin (red) and DNA (DAPI staining, blue). Bar 5 μm.

To explore further possible chromosome endoreduplication, in addition to traditional methods of chromosome visualization, we used immunofluorescence and high resolution wide-field microscopy method for centromere-associated protein CENP-A detection and quantification in interphase cells (Figure 4A,C). When comparing the interphase nuclei after actin’s depletions had the impression to change their sizes. To determine the nuclear size after β- and γ-actin depletion, we have measured the area of the nucleus. Morphometrical analysis showed that the average nuclear area of β-actin-suppressed cells was increased compared with sh-control, whereas downregulation of γ-actin caused a significant reduction of the nuclear area (Figure 4B). The quantification of CENP-A-containing nucleosome particles showed the difference of MDA-MB-231 cell populations in both β- and γ-actin suppressed cells compared with control (Figure 4D). Control population display nearly normal distribution histogram with modal class 41–55 of CENP-A nucleosome particles/points per cell (Figure 4E). In β-actin-suppressed MDA-MB-231, besides “control-like” cells with modal class 51–60, another population with a number of CENP-A points exceeding 60 was identified. It is noteworthy that in γ-actin downregulated MDA-MB-231, prevailed cells with half number of CENP-A points per cell compared with control (Figure 4E).

Phospho-Histone H3 is an immunomarker specific for mitosis. Histone H3 phosphorylation may initiate at different mitotic phases, but metaphase chromosomes are always found to be highly phosphorylated. pH3 staining is a useful method for determining proliferative potential [16]. The protein expression levels of CENP-A, phospho-histone H3 (pH3), and histone H3 (H3) were determined by western blot (Figure 4F,H). Silencing of β-actin in MDA-MB-231 cells, accompanied by increased expression of CENP-A protein, with the attendant augmentation the number of cells with a large quantity of CENP-containing nucleosome particles in interphase, suggests an increase of endoreduplication. Downregulation of γ-actin showed significant reduction of CENP-A and pH3 expression compared with sh-control, which may reveal a decrease in reduplication process. The decrease of cell fraction with number of CENP-A-containing nucleosome particles exceeding 46 also indicates this effect. 

We performed the IF staining with an anti-H3 antibody (Figure 4G) and an anti-pH3 antibody, a marker of the mitotic phase (Figure 4I). We confirmed that interphase nuclei did not stain with pH3. Downregulation of γ-actin showed practically total reduction of pH3-positive cells, which confirms a decrease in the frequency of mitotic cells revealed by IF cytometry. We have measured the IF intensity of pH3 for the staining in the mitotic chromosomes and revealed that IF was different depending on the stage of mitosis (displayed in Figure S2). Actin depletion did not influence pH3 IF compared to control cells. We suppose that a decrease of pH3 in γ-actin depleted cells shown by Western blot occurs due to several factors: (1) an increase in the number of interphase cells and a proportional decrease of mitosis (see Figure 2), (2) an increase in rate of telophase cells, in which pH3 IF intensity is significantly reduced. 

## 3. Discussion

We have previously shown that the relative level of β-actin was decreased in tumors compared with corresponding normal tissues (cervical, breast, colon, and lung), while γ-actin was expressed evenly and diffuse in all studied normal and malignant tissues [5,17,18,19]. We have also shown that β- and γ-actins have distinct roles in tumorigenesis [5]. It should be noticed that the ratio between isoforms and reciprocal regulation of actins amount on both an mRNA and protein level (downregulating of one actin isoform stimulates increase of another isoform) might be responsible for the observed effects [5,10,11,12,13]. Our previous study of mitotic MCF-7 cells showed that downregulation of β-actin led to an enrichment of prophase/metaphase population compared with control, whereas γ-actin downregulation induced telophase enrichment. This work demonstrates that β-actin depletion leads to an obvious increase in the number of cells in G2/M phase and to an enrichment of aneuploidy in this cell population compared with parental hypo-triploid MDA-MB-231 cells. MDA-MB-231 cell line derived from basal-like and triple-negative breast cancer (TNBC), representing a clinically aggressive subtype of breast cancer with poor outcomes [20,21]. Chromosomal instability (CIN) is a feature of TNBC and many other types of cancer, and it results from errors in DNA replication, DNA repair, or chromosomal mis-segregation [22]. CIN is of particular interest for experimental and practical oncology because it is associated with aggressive tumors, the acquisition of drug resistance, and poor patient prognosis [23]. The treatment with antimitotic drugs increased chromosome numbers in cancer cells significantly [24,25]. Our results correlate with previous observations showing that antimitotic drugs may induce mitotic slippage—Completion of mitosis without cytokinesis. The surviving cells after mitotic slippage become tetraploid. It was shown, that reinforcing mitotic arrest with inhibitors of mitotic slippage leads to increased cell survival and proliferation, whereas inducing of mitotic slippage leads to prolonged cell death [26]. Nuclear alterations characterizing the mitotic catastrophe after mitotic slippage provide increased interest in the development of new methods of anticancer therapy [27].

We noted an enhancement of endoreduplication after β-actin depletion in MDA-MB-231 cells, which can contribute to genome instability [24]. Deceleration of this process in cancer drug-treated cells could reduce the oncogenic potential of highly aggressive malignancies. It is important to emphasize that γ-actin downregulation decreased chromosome numbers accompanied by telophase enrichment and cell cycle arrest in G1 phase. Hopefully, by using the right way of mitotic arrest, we could find a tumor cells-suppressing strategy of chromosomal instability reduction resulting in minimized long-term growth of TNBC cancer cells. 

Centromere-associated proteins such as CENP-A are essential regulators for chromosomal stability. Overexpression of CENP-A could be the reason of chromosomal instability [28,29,30,31], so CENP-A overexpression have been observed in several cancers, and this situation correlated with poor prognosis [32,33,34,35,36]. Here we discovered that γ-actin downregulation significantly reduced CENP-A and pH3 expression, as well as the number of CENP-A-containing nucleosome particles, indicating a decrease in reduplication process.

Previously, we have shown that there is no effective inhibition of proliferation with a decrease in the expression of β-actin in MDA-MB-231 breast cancer cells. It is possibly connected with mutation in p53 gene. We have shown that a shift in the ratio of actin isoforms towards a decrease of β-actin in the tumor cell lines HCT116, A549 and, MCF-7 led to inhibition of the cell cycle and cell death. However, in cells with mutations in p53 gene, as MDA-MB-231, or other disorders of p53 [37], there was no cell cycle suppression. These cell types may generate polyploidy and accumulated genetic changes leading to tumorigenesis. Concerning our data are the results on normal mammalian oocytes. An unexpected actin-dependent mechanism that drives the accurate alignment and segregation of chromosomes in mammalian eggs was recently reported [38]. Authors disrupted spindle actin by two different approaches: isolation of oocytes from Fmn2-/- mice, in which lack of the actin nucleator formin-2 disrupts spindle actin, as well as treatment of oocytes with the actin-depolymerizing drug cytochalasin D. It was also shown that the mitotic furrow is specifically assembled from β-actin filaments by the actin nucleator formin DIAPH3 at the site of cytokinesis [39]. It is worth mentioning that β-actin bundles are more sensitive to the treatment with cytochalasin D compared with γ-actin structures [3]. Furthermore, our recent data revealed microtubule-dependent transfer of β-actin polymers to the cortex during metaphase [4]. On the contrary, we show here that γ-actin downregulation results not only in reduced percentage of mitotic carcinoma cells, but leads to chromosome stability, reduced polyploidy, and aneuploidy, which is especially important for cancer targeting. The above results once again prove the functional diversification of non-muscle actin isoforms not only in normal, but also in tumor cells. A growing body of evidence suggests that aneuploidy is often caused by chromosomal instability, which may reflect defects in mitotic segregation in cancer cells. Proper insight into the molecular mechanisms leading to aneuploidy holds promise for the development of cancer drugs that target this process [7].

## 4. Materials and Methods

### 4.1. Cell Culture

For the study, we used human breast cancer MDA-MB-231 cell line (ATCC^®^ HTB-26^™^). Cells were maintained in Dulbecco’s modified Eagle media (DMEM- high glucose, 01-052-1A, Biological Industries, Beit HaEmek, Israel) with 5% Certified Fetal Bovine Serum (04-001-1A, Biological Industries, Beit HaEmek, Israel) and 4mM glutamic acid at 37 °C in the atmosphere of air containing 5% CO_2_.

### 4.2. DNA Constructs

Previously selected and verified [5] β- and γ-actins specific shRNA-expressing constructs were used. Briefly, for β- and γ- actins sh-dependent repression 21 nt target sequences 5′-CAAATATGAGATGCGTTGTTA-3′ corresponding to 1465-1475 of β-actin mRNA ref|NM_001101.3| and 5′-CAGCAACACGTCATTGTGTAA-3′ corresponding to 2057-2077 of γ-actin mRNA ref|NM_001199954.1 included in hairpin structures were cloned into pLKO.1 (Sigma-Aldrich, St.Louis, MO, USA) lentiviral vector. pLKO.1-shGFP-puro targeting eGFP (GenBank Accession No.pEGFP U55761) was used as a control. To prove of β- and γ- actins mRNAs repression caused by sh-RNA expression, total mRNA was isolated with SV Total RNA Isolation System (Promega) according to the manufacturer’s protocols and mRNA amounts of β- and γ-actin by routine PCR were tested. The following primers were used: β-actin forward 5′-ACAGAGCCTCGCCTTTGC-3′, reverse 5′GAGGCGTACAGGGATAGCAC-3′; γ-actin forward 5′-CAAAAGGCGGGGTCGCAA-3′, reverse 5′-TGGGGTACTTCAGGGTCAGG-3′; α-tubulin forward 5′GTTGGTCTGGAATTCTGTCAG-3′, reverse 5′-AAGAAGTCCAAGCTGGAGTTC-3′. The quantification of mRNA bands was performed using Chemi-Smart 3000 Imaging System (Vilber Lourmat, Collégien, France) and TotalLab v.2.01 software (data not shown). Oligonucleotides synthesis and DNA sequencing were performed by Evrogen (Moscow, Russia). Further, actin isoforms specific repression was tested by western-blot analysis.

### 4.3. Viral Transfection and Cell Infection

pLKO.1 lentiviral DNA constructs together with the pΔR8.2, (#12263, Addgene, Watertown, MA, USA) and pVSV-G, (#8454, Addgene, Watertown, MA, USA) packaging plasmids were transfected into 293FT packaging cells (R70007, Invitrogen, Carlsbad, CA, USA) using GeneJuice Transfection Reagent (70967, Sigma-Aldrich, St. Louis, MO, USA). Virus-containing supernatants were collected every 12 h during 3 days after transfection and used to infect the recipient cells in the presence of 8 μg/mL polybrene (TR-1003-G, Sigma-Aldrich, St. Louis, MO, USA). Infected MDA-MB-231 cell cultures infected with pLKO-based lentiviruses were selected during 5 days in the medium containing 2,5 µg/mL puromicin (P8833, Sigma-Aldrich, St. Louis, MO, USA) for pLKO.1-puro constructs. All experiments were performed 6 days after vector-mediated gene transfer.

### 4.4. Cell Cycle Analysis

After 6d of β- or γ-actin depletion by corresponding shRNAs MDA-MB-231 cells were collected for cell cycle analysis. The cells were harvested, washed with PBS, and fixed with ice-cold 80% ethanol, incubated overnight at 4 °C and stored at 4 °C until analyzed by flow cytometry. Briefly, the cells were spun down to remove the ethanol, washed with PBS and stained with 30 µg/mL Propidium Iodide (MP Biomedicals, Strasbourg, France) and 10 ng/mL RNAse A (Fermentas, Vilnius, Lithuania) in PBS for 45 min at 37 °C in the dark. Cells were analyzed using Beckman Coulter Cytomics FC500 flow cytometer and cell cycle distribution was analyzed with ModFit LT (Verity Software House) software.

The quantitative ratio of cells in different mitotic phases was analyzed by cellular staining for DNA by DAPI. Different mitotic phases were observed by microscopic analysis by scoring 300 cells for each condition. 

### 4.5. Analysis of Chromosomal Aberrations

MDA-MB-231 cells were plated on dishes and incubated for 2 days. Incubation with tubulin-destabilizing drug colcemid (Demecolcine; D1925, Sigma-Aldrich, St. Louis, MO, USA, final concentration 0.05 µg/mL) in pre-warmed medium for 12 h and subsequent release into the fresh medium were used to increase the population of cells in metaphase. The detached by trypsinization cells were incubated in the warm hypotonic solution (0.075 M KCl, 15 min, 37 °C), and preserved with Carnoy’s fixative (3:1 ratio of methanol:glacial acetic acid, 30 min, at ice). Fixed cells were dropped onto the glass slides, air-dried properly and: (1) stained by Giemsa staining solution (3:1 ratio of Gurr’s Buffer and Giemsa Stain, 5 min, room temperature) and rinsed with distilled water to obtain unbanded stained chromosomes; (2) incubated with 0.025% trypsin working solution for 10 sec with following Giemsa staining (3:1 ratio of Gurr’s Buffer and Giemsa Stain, 10 min), and distilled water rinsing. Unbanded chromosome staining was performed on 200 metaphase chromosomal preparations for each condition. GTG-staining was performed on 20 metaphase control samples. Chromosomes on the obtained samples were examined by Olympus BX41 microscope with 10x and 100x oil immersion lenses.

### 4.6. Western Blot Analysis

Whole cell extracts were lysed in ice-cold RIPA buffer (50 mM Tris-HCl pH 7.4, 150 mM NaCl, 1% deoxycholate Na, 1% NP-40, 0.1% SDS, 100 mM PMSF, 1 mM pepstatin A and 1 mM E64). Protein concentration in the extracts was determined with a protein assay system (BioRad, Hercules, CA, USA). 5–20 µg of protein was separated on 8–12% SDS polyacrylamide gel and transferred to PVDF membrane (IPFL00010, Millipore, Burlington, MA, USA). The membranes were blocked with SuperBlock Blocking Buffer (Thermo Scientific, Waltham, MA, USA) and then probed with antibodies specific to corresponding proteins. Membranes were treated with Alexa488-conjugated secondary antibodies (A11029, Invitrogen, Carlsbad, CA, USA), band detection was performed using variable mode imager Typhoon9410 (GE Healthcare, Chicago, IL, USA). Signals of β-actin, γ-actin, CENP-A, H3, and pH3 were quantified by densitometry and normalized using total actin signal as reference protein. The difference in the relative amount of protein was estimated on the results of at least three independent experiments.

### 4.7. Antibodies

Mouse monoclonal antibodies to: β-actin (MCA5775GA, AbD Serotec); γ-actin (MCA5776GA, AbD Serotec, Raleigh, NC, USA); pan actin (4968, Cell Signaling) were used. Rabbit polyclonal antibodies to CENP-A (2186, Cell Signaling) were used. Rabbit monoclonal antibodies to: Phospho-Histone H3 (Ser10, 3377, Cell Signaling); Histone H3 (4499, Cell Signaling) were used.

The following secondary antibodies were used: AlexaFluor488-, AlexaFluor594- conjugated goat anti-mouse IgG, IgG1, IgG2b and goat anti-rabbit IgG (Jackson ImmunoResearch Laboratories Inc, West Grove, PA, USA). 

### 4.8. Immunofluorescence Microscopy

Cells were grown on glass coverslips, rinsed with DMEM containing 20 mM Hepes and 5 mM MgCl_2_ at 37 °C, fixed in 2% PFA in DMEM/Hepes/5 mM MgCl_2_ at 25 °C for 10 min and treated for 5 min with MeOH at −20 °C. Cells were incubated with primary and secondary antibodies. DAPI (D9542, Sigma-Aldrich, St. Louis, MO, USA) was applied into secondary antibodies for nuclear staining. Immunofluorescence was observed using Axioplan microscope with 100 ×/1.3 Plan-Neofluar lens (Carl Zeiss, Jena, Germany). The quantification of nuclear area as projection on the substrate (outlines of nuclear regions) were performed on the DAPI-stained nuclei. The nuclear area and CENP-A dots were analyzed with ImageJ 1,52v Software (NIH, Bethesda, MD, USA). Immunofluorescence (IF) intensity of pH3 was analyzed with the ImageJ 1,52v Software using the “freehand selection” tool in a single-color channel. Mean values of IF intensity for chromosome regions measurement were obtained for each cell. The results are presented as mean for 15 cells ± standard error of the mean for each mitotic phase in each condition.

### 4.9. 3D-IF Microscopy (Wide-Field with Deconvolution)

Samples were examined using a Nikon N-SIM (Nikon, Tokyo, Japan) with 100x/1.49 NA oil immersion objective, 488 nm and 561 nm diode laser excitation. Image stacks (z-steps of 0.12μm) were acquired with EMCCD camera (iXon 897, Andor, effective pixel size 60 nm). Exposure conditions were adjusted to get a typical yield about 5000 max counts (16-bit raw image), while keeping bleaching minimal. Serial optical sections of the same cell taken in wide-field mode were deconvolved using the AutoQuant blind deconvolution algorithm. Image acquisition and data alignment were performed using NIS-Elements 4.2 software (Nikon, Tokyo, Japan).

### 4.10. Statistical Analysis

Results are presented indicating mean ± standard error of the mean of at least three independent experiments. Intergroup differences were analyzed by the Mann–Whitney U test or Student’s t-test. Three independent experiments are marked as n = 3 in the figure legends. Error bars at the graphs represent SEM. Values of *p* < 0.001 (***), *p* < 0.01 (**), and *p* < 0.05 (*) were considered as statistically significant.

## Data Availability

The data presented in this study are available on request from the corresponding author.

## Supplementary Materials

The following are available online, Figure S1: Karyotypic analysis of MDA-MB-231 cell line, Figure S2: Immunofluorescence (IF) intensity of pH3 histone in mitotic MDA-MB-231 control cells and with down-regulated β- or γ-actin.

## Sample Availability

Samples are not available.
