# Supplementary material for: Impaired Expression of Cytoplasmic Actins Leads to Chromosomal Instability of MDA-MB-231 Basal-Like Mammary Gland Cancer Cell Line
Source: Molecules. 2021 Apr 8;26(8):2151. doi: 10.3390/molecules26082151 (PMC8068389; doi:10.3390/molecules26082151)
Supplement: Supplementary file 1 [file molecules-26-02151-s001.pdf]

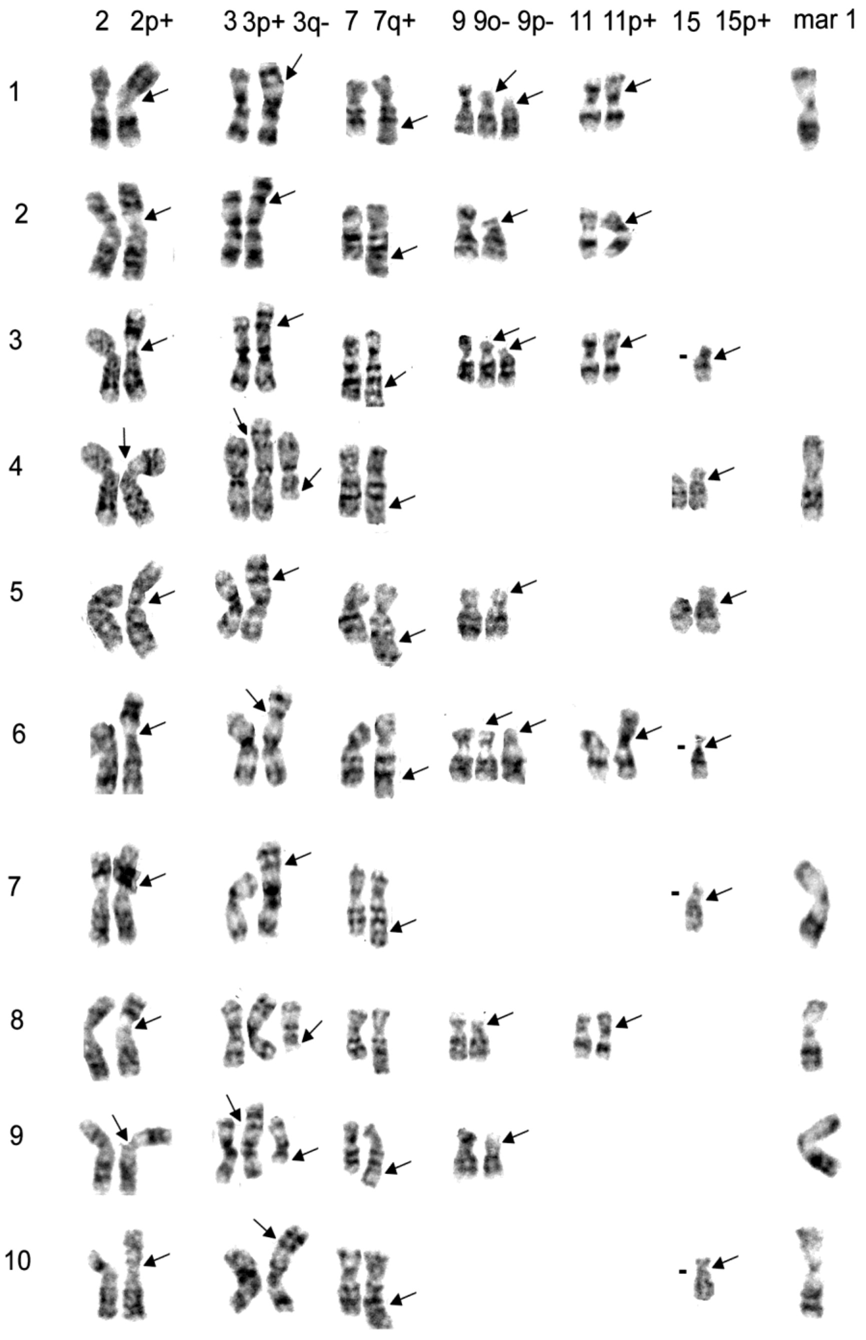

Figure S1. Karyotypic analysis of MDA-MB-231 cell line.

Clonal chromosome markers and its normal homologs from ten karyotyped cells of MDA-MB-231 cell line. Arrows point to main chromosomal alterations. Giemsa staining (G-banding).

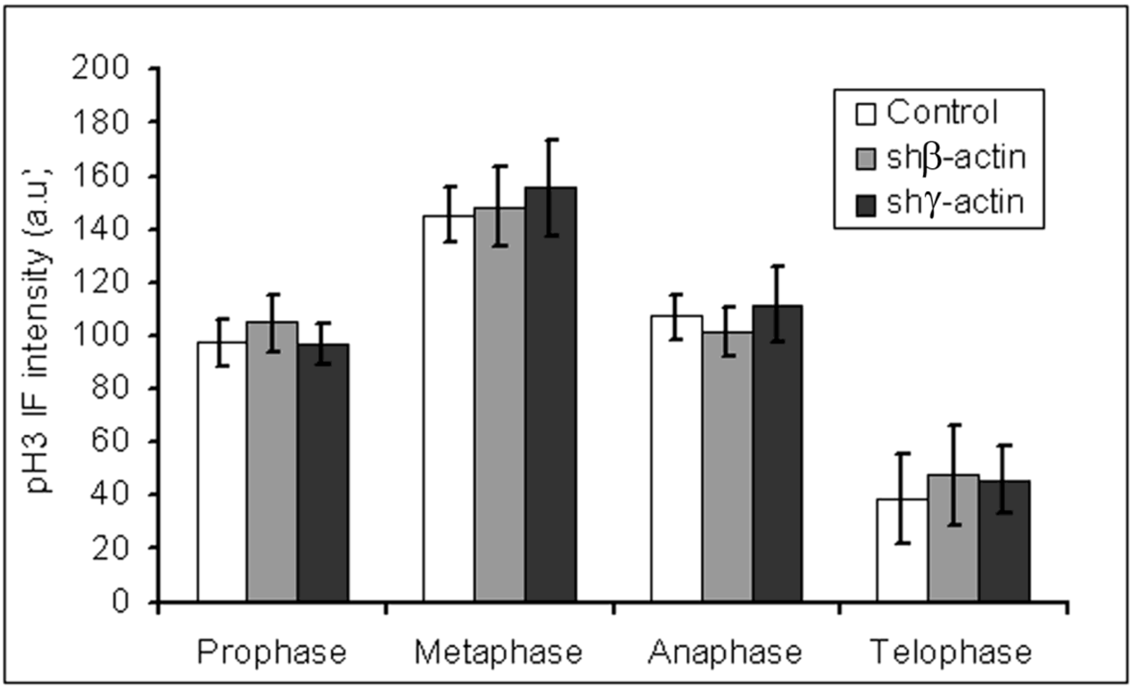

Figure S2. Immunofluorescence (IF) intensity of pH3 histone in mitotic MDA-MB-231 control cells and with down-regulated β- or γ-actin.

Graph represents the quantification of IF- pH3 intensity of chromosome area in MDA-MB-231 cells with down-regulated β- or γ-actin (Mean ± SEM, n=15 for each phase and condition of actin expression) at the different mitotic phases.
